# A Moderated Mediation Model of Mentoring and Coaching and Quiet Quitting Behaviour: The Mediating Role of Work Engagement and the Moderating Role of Job Insecurity

**DOI:** 10.3390/bs16050829

**Published:** 2026-05-21

**Authors:** Samuel Siwela, Cebile Tebele

**Affiliations:** Department of Industrial and Organisational Psychology, University of South Africa, Pretoria 0002, South Africa

**Keywords:** graduate interns, early career employees, mentoring and coaching, work engagement, quiet quitting, job insecurity

## Abstract

Quiet quitting is reported to be on the increase in the post-COVID pandemic workplace, especially among early-career Generation Z (Gen Z) employees. This trend poses serious challenges and could negatively affect organisational productivity, performance, and profitability. The purpose of this study is to evaluate the effects of mentoring and coaching on quiet quitting among graduate interns. This study also seeks to assess whether work engagement mediates this relationship and job insecurity moderates the mediated relationship between mentoring and coaching and quiet quitting via work engagement. Purposive and snowball sampling were used to recruit participants. Data were collected from 264 graduate interns employed in fixed-term internship programmes in South African organisations. The data was analysed using the SPSS PROCESS macro and SPSS Amos 30 graphics. The results showed that mentoring and coaching was significantly and negatively related to quiet quitting behaviours among graduate interns, and this negative relationship was partially mediated by work engagement. Furthermore, job insecurity moderated the mediated effect of mentoring and coaching on quiet quitting behaviours via work engagement. This study advances our understanding of how organisations can mitigate quiet quitting among graduate interns by integrating the social exchange theory and Job Demands–Resources model (JD-R). The practical implication for organisations is to capacitate line managers with technical, professional, and interpersonal skills to mentor and coach early-career Gen Z employees. Line manager mentoring and coaching will increase early-career Gen Z employees’ work engagement and subdue quiet quitting, which is reported to be on the rise among this generation.

## 1. Introduction

Quiet quitting is becoming a serious concern in organisations. Quiet quitting describes the intentional behaviour of employees who limit their effort, contribution, and refrain from going the extra mile in pursuit of organisational goals. Quiet quitting does not imply that an employee leaves the organisation; rather, it reflects an intentional decision to limit effort and contributions to doing just enough to remain employed (Anand et al., 2024). Recent surveys seem to suggest that quiet quitting is prevalent among employees (Deloitte, 2025; Gallup, 2023). This is also corroborated by Gallup (2023), who indicated that 59% of employees are not engaged and 18% are actively disengaged, and these are prone to engaging in quiet quitting behaviours. In the healthcare context, a review by Burch et al. (2025) found that a lack of employee recognition, high job demands, and inadequate organisational support led to employee burnout, which in turn led to quiet quitting. Moreover, a study by Galanis et al. (2024) found that 60% of nurses engaged in quiet quitting behaviours.

This study targets graduate interns, a subset of Generation Z (Gen Z), not the entire Gen Z workforce. Graduate interns aged 21–26 constitute a subgroup of the Gen Z cohort, born between 1995 and 2010 (Xueyun et al., 2023). The graduate interns who were the target of this study constitute a subgroup of the entire cohort and represent early-career Gen Z employees. Therefore, graduate interns will be used interchangeably with early-career Gen Z employees to reflect this subgroup and separate it from the entire Gen Z cohort. In this study, Gen Z refers to the entire cohort, while early-career Gen Z refers to the subgroup of graduate interns. Research has reported that quiet quitting is highly prevalent among Gen Z employees (Oktavia et al., 2025). Hamouche et al. (2023, p. 4297) substantiated this by noting that “quiet quitting mostly resonates with young employees due to the drastic changes following the COVID-19 pandemic”. This is also amplified by the 2025 Deloitte Gen Z and Millennial survey that noted that “with the emergence of viral trends such as ‘quiet quitting’, ‘bare minimum Mondays’, and ‘productivity theater’, the Gen Zs have gained a reputation for not putting effort at work” (Deloitte, 2025, p. 7). This is further exacerbated by the volatility, uncertainty, complexity, and ambiguity (VUCA) of the modern workplace, which has augmented employee anxiety, stress, and emotional exhaustion. Hence, Gen Z has been termed the anxious generation (Oktavia et al., 2025) and is reported to be more prone to quiet quitting tendencies (Ochis, 2024; Srivastava & Saxena, 2025; Taufik et al., 2024). This presents a huge challenge for organisations since quiet quitting has been linked to negative outcomes such as decreased individual- and organisational-directed citizenship behaviours (Patel et al., 2025), decreased employee and organisational performance (Gone et al., 2026; Liu-Lastres et al., 2024), high turnover intentions (Galanis et al., 2024; J. S. Kim et al., 2024; Othman et al., 2025), lower levels of customer service (Liu-Lastres et al., 2024), and lower levels of commitment and loyalty (Othman et al., 2025).

Recent research has focused on the quiet quitting of Gen Z employees (Nguyen & Vu, 2025; Ochis, 2024; Srivastava & Saxena, 2025; Taufik et al., 2024; Xueyun et al., 2023). Most studies have investigated the trigger factors or antecedents of quiet quitting (Alhammadi et al., 2025; Gone et al., 2026; Karrani et al., 2025; Nguyen & Vu, 2025; Srivastava et al., 2024; Taufik et al., 2024; Xueyun et al., 2023) and the impact of quiet quitting (J. S. Kim et al., 2024; K. T. Kim & Sohn, 2024; Gone et al., 2026), but few studies have focused on the factors that can mitigate the manifestation of employee quiet quitting behaviours (Burch et al., 2025; Karrani et al., 2025). A study by Karrani et al. (2025) showed that relational job design was negatively associated with quiet quitting behaviours, while Burch et al. (2025) showed that authentic leadership strategies fostered a supportive and engaging work environment, which was negatively associated with quiet quitting behaviours.

However, these studies emphasise the direct relationship between organisational practices and withdrawal behaviour. There is limited attention on the underlying theoretical mechanism through which organisational practices are negatively related to quiet quitting. For example, the study by Burch et al. (2025) indicated that authentic leadership strategies reduce quiet quitting, but it did not provide an empirically tested theoretical mechanism underlying this effect. There is a need for studies that provide theoretical mechanisms through which organisational practices, such as authentic leadership strategies and mentoring and coaching practices, influence withdrawal behaviours. This study addresses this gap by integrating the Job Demands–Resources (JD-R) model (Demerouti et al., 2001) and social exchange theory (SET) (Cropanzano & Mitchell, 2005) to investigate the impact of mentoring and coaching on graduate interns’ quiet quitting behaviours. The JD-R model proffers a structural framework that posits that mentoring and coaching are developmental job resources that primarily drive work engagement through activating the motivational process, which in turn is negatively associated with withdrawal behaviours. In the same vein, mentoring and coaching organisational practices are interpreted as a non-monetary social exchange inducement, which activates the norm of reciprocity, and the reciprocation manifests as higher levels of work engagement, which counteract the occurrence of withdrawal behaviours. The JD-R model explains why development resources, such as mentoring and coaching, drive work engagement by activating the motivational process, while the social exchange theory explains the psychological mechanism by which developmental resources increase work engagement through the norm of reciprocity. Thus, integrating JD-R and social exchange theory is generative because it advances our understanding of how the two theories complement one another to explain how developmental job resources are associated with work engagement, which, in turn, is negatively associated with withdrawal behaviours.

Therefore, utilising the JD-R model (Demerouti et al., 2001) and social exchange theory (SET) (Cropanzano & Mitchell, 2005), this study aims to investigate the impact of mentoring and coaching on graduate interns’ quiet quitting behaviours and whether work engagement mediates this relationship. Given that internship employment is precarious and associated with high job insecurity, the question of whether job insecurity serves as a contextual moderator, strengthening or weakening the indirect effect of mentoring and coaching on quiet quitting, has not been adequately tested empirically. This study fills this gap by using the JD-R boosting hypothesis to propose and empirically test the proposition that perceived job insecurity among graduate interns strengthens the indirect effects of mentoring and coaching on quiet quitting. This study further contributes to the literature on factors that mitigate quiet quitting among graduate interns or early-career Gen Z employees by investigating how line manager mentoring and coaching can be a powerful developmental intervention that increases work engagement and subdues the rise in quiet quitting tendencies among graduate interns.

## 2. Theoretical Background and Hypothesis Development

### 2.1. Quiet Quitting

Quiet quitting is a construct described as “an old wine in new bottles” because it has been introduced as a contemporary construct, though it is linked to previous constructs such as employee disengagement and withdrawal behaviour (Atalay & Dağıstan, 2024). Employee disengagement refers to a psychological state in which employees physically, cognitively, and emotionally distance themselves from the job and can manifest as a lack of involvement and interaction with colleagues (Afrahi et al., 2022; Atalay & Dağıstan, 2024). It can arise when job demands exceed available coping resources (Bakker & Demerouti, 2007). Withdrawal behaviours on the other hand, refer to observable behaviour such as withdrawal effort, neglecting tasks, arriving late, leaving work early, and taking long breaks (Lehman & Simpson, 1992). Quiet quitting reflects employee disengagement and psychological detachment; however, employees who engage in quiet quitting invest effort only to achieve the bare minimum (Liu-Lastres et al., 2024; Serenko, 2024). This indicates that quiet quitting does not imply complete detachment or disengagement on the part of employees. Quiet quitting behaviour does not equate to complete detachment or disengagement but rather captures how the employee reduces his/her investment in time and effort to meet minimum performance expectations or deliverables (Hamouche et al., 2023). Bernuzzi et al. (2025, p. 11004) defined quiet quitting as a “work-related tendency where employees do not formally resign but intentionally limit their work engagement to the minimum required duties”. This implies that the employee voluntarily reduces work effort and contribution to the organisation. In the long run, the employee becomes psychologically, emotionally, and physically withdrawn; hence, quiet quitting behaviour is regarded as a psychological and behavioural withdrawal construct (Jalali et al., 2026).

The prevalence of quiet quitting among Gen Z employees presents a serious challenge to contemporary organisations. Gen Z employees are entering the workforce in large numbers and are projected to become the largest generation by 2050 (Xueyun et al., 2023). Mentoring and coaching can be viable interventions to increase work engagement and address the rise in quiet quitting behaviours among early-career Gen Z graduate interns. A recent study by P. S. Ngobeni et al. (2026) found that supportive line managers facilitated positive psychosocial experiences among early-career Gen Z employees, which is likely to be positively associated with work engagement and negatively associated with quiet quitting. This emphasises the important role that effective mentoring and coaching can play in increasing work engagement and reducing quiet quitting behaviours among early-career Gen Z graduate interns.

### 2.2. Mentoring and Coaching

In this study, mentoring and coaching are combined into a single construct, but this does not imply that the two constructs are conceptually distinct. For example, mentoring emphasises long-term development, psychosocial support, counselling, and role modelling, while coaching emphasises short-term skills, more targeted development, and performance feedback (D’Abate et al., 2003). Mentoring and coaching exhibit a conceptual overlap and, within organisational settings, may be used concurrently, which can sometimes blur the distinction between them. As noted by Weinberg (2024, p. 630), “coaching often appears in many mentoring studies as a characteristic of mentoring’s career support”. Therefore, the conceptual overlap makes it plausible to combine the two constructs into a single construct capturing developmental job resources, which aligns with the Job Resources–Demands (JD-R) model. As developmental job resources, mentoring and coaching are “functional in achieving work goals; reduce job demands with the associated physiological and psychological costs; and stimulate personal growth, learning, and development” (Demerouti et al., 2001, p. 501). Mentoring and coaching activate the motivational process leading to high engagement (Bakker & Demerouti, 2007). Previous research has conceptualised mentoring and coaching as job resources (DuPlessis et al., 2021; Garg et al., 2021; Lin et al., 2021). Furthermore, studies have combined functionally similar job resources into a composite construct (Tadić et al., 2015; Trépanier et al., 2014; Uslukaya & Zincirli, 2025). Therefore, combining mentoring and coaching as a single construct to capture developmental job resources aligns with the level of analysis at which the JD-R framework is applied.

Mentoring and coaching experiences in this study refer to the relationship between an experienced line manager and a novice graduate intern, in which the line manager provides support, direction, feedback, and mentoring that are linked to the graduate’s skills development, performance, and career development (Kram, 1985). Mentoring and coaching experiences foster the graduate intern’s personal development and are likely to increase their satisfaction with the internship programme. High-quality mentoring support and coaching received from the line manager is likely to enhance the graduate intern’s self-esteem (Ghosh et al., 2012; Sloan et al., 2025), enhance self-efficacy (Moon et al., 2025), facilitate the development of the psychological capital (Carter & Youssef-Morgan, 2019), and reduce depressed mood at work (L. T. Eby et al., 2010). Furthermore, receiving mentoring and coaching from the line manager fosters a trusting relationship that is important in helping the graduate intern deal with job and career-related uncertainties while promoting work engagement, organisational commitment, and the well-being of graduate interns (Chun et al., 2012; Fowler et al., 2021; Sloan et al., 2025). Mentoring and coaching also lead to the acquisition of skills and knowledge, which improve the graduate’s performance and future employability (Bozionelos et al., 2016; Niu et al., 2025).

Furthermore, line manager mentoring and coaching provide a supportive work environment that facilitates the transition of early-career Gen Z employees into the world of work. Managerial coaching is very important for entry-level Gen Z graduate interns because it provides an experiential learning mechanism and a useful developmental intervention that facilitates skills acquisition, development, and performance (Ellinger et al., 2011; McCarthy & Milner, 2013). Therefore, mentoring and coaching graduate interns are important to ensure they are highly engaged and adequately supported as they transition to the world of work and face early-career challenges. Managerial coaching is commonly positioned in the literature as relevant to work engagement (Tanskanen et al., 2019; L.-y. Wang et al., 2024), psychological safety, self-efficacy (Q. Wang et al., 2022), well-being (Zhao & Liu, 2020), in-role and extra-role behaviours (J. T. Huang & Hsieh, 2015; S. Kim & Kuo, 2015), job satisfaction, performance (S. Kim, 2014; Pousa et al., 2017), and organisational commitment (S. Kim, 2014). Thus, managerial coaching is particularly valuable for graduate interns who may lack confidence, work experience, and practical skills, as it supports their development and helps them adjust to early-career workplace demands.

Hence, there has been a call for professionally trained, interpersonal-competent mentors to mentor graduate interns (Mseleku, 2024; Pop et al., 2013). Research has shown that some graduate intern mentors lack requisite skills and competencies, which have been associated with negative mentoring experiences among graduate interns (Mchunu & Mutereko, 2020; Mseleku, 2024; T. Ngobeni et al., 2025). Negative mentorship experiences can be caused by mentee–mentor mismatch, mentor unavailability, lack of interest in mentoring the mentee, manipulative mentor behaviour, lack of trust between mentee and mentor, and the mentor’s lack of technical, professional, and interpersonal skills (L. Eby et al., 2004; L. T. Eby & McManus, 2004; Mseleku, 2024; Pinho et al., 2005). Furthermore, bad mentoring experiences have been observed to co-occur with mentees’ depressed mood at work and psychological withdrawal (L. T. Eby et al., 2010). Therefore, managers of graduate interns need to be trained to become competent mentors, which emphasises the importance of their ability to mentor and coach their interns.

### 2.3. Research Model and Hypotheses Development

The JD-R provides a structural mechanism through which developmental job resources, such as mentoring and coaching, influence work engagement and withdrawal behaviours. Developmental job resources refer “to those, psychological, social, or organisational aspects of the job that stimulate personal growth, learning, and development” (Bakker & Demerouti, 2007, p. 312). The JD-R model posits that developmental job resources such as mentoring and coaching are positively associated with work engagement (Bakker & Demerouti, 2007). As developmental resources, mentoring and coaching are likely to provide psychosocial and career development support that will directly affect work engagement. The JD-R model explains how developmental resources activate the motivational process, thereby increasing work engagement, which in turn is negatively associated with quiet quitting behaviours. This is supported by F. Yang et al. (2022), who found that mentoring positively affected proteges’ work engagement. In a similar vein, a study by Z. Wang et al. (2018) also indicated that supervisor mentoring positively influenced a new employee’s work engagement. On the other hand, the JD-R model also posits that job resources, such as mentoring and coaching, are negatively associated with quiet quitting because they buffer work demands and resource depletion, thereby undermining tendencies to engage in withdrawal behaviours. Previous research has shown that mentoring is negatively associated with withdrawal behaviours (Firzly et al., 2022; Garg et al., 2021; S. S. Kim et al., 2015).

The social exchange theory (SET) (Cropanzano & Mitchell, 2005), on the other hand, complements the JD-R model and explains how developmental job resources influence work engagement and quiet quitting behaviours by explaining why employee developmental practices influence work engagement and quiet quitting. Within social exchange theory, the developmental practices of mentoring and coaching are interpreted as organisational inducements in the social exchange relationship, activating the norm of reciprocity. In line with social exchange theory (Cropanzano & Mitchell, 2005), this reciprocation is associated with positive work attitudes and behaviours, which are positively associated with work engagement and negatively associated with quiet quitting. Previous research has shown that the effective mentoring and coaching of graduate interns is likely to cultivate positive work attitudes and behaviours such as work engagement (Ladyshewsky & Taplin, 2018; F. Yang et al., 2022) and organisational citizenship behaviours (Chang & Uen, 2022) and is negatively associated with withdrawal behaviours such as intention to quit, absenteeism, and quiet quitting (L. Eby et al., 2004; Garg et al., 2021; C. Yang et al., 2019). In contrast, lack of mentoring and coaching during the internship may be construed as an unfavourable exchange relationship that can have detrimental effects on the intern’s development, skills acquisition, and knowledge (Mchunu & Mutereko, 2020). This unfavourable exchange relationship can trigger withdrawal and quiet quitting behaviours, which manifest through the graduate intern doing only what is enough to meet the minimum required deliverables. This is supported by previous studies that have shown that negative mentoring experiences are associated with depression and psychological withdrawal (L. Eby et al., 2004). Therefore, mentoring and coaching are expected to be positively associated with work engagement and negatively associated with quiet quitting behaviours among graduate interns. Thus, the following hypotheses are formulated:

**H1.**  *Perceived mentoring and coaching is negatively related to quiet quitting behaviours of graduate interns.*

**H2.**  *Perceived mentoring and coaching is positively related to work engagement of graduate interns.*

### 2.4. Work Engagement

Work engagement is defined as “a positive, fulfilling, work-related state of mind that is characterised by vigor, dedication and absorption” (Schaufeli et al., 2002, p. 74). Vigour and dedication are considered the core dimensions of work engagement (Bakker & Demerouti, 2007). Vigour refers to an individual’s high energy levels and mental resilience in the face of challenging work situations (Schaufeli et al., 2002). Dedication, on the other hand, refers to an employee’s feelings of inspiration, enthusiasm, and strong involvement in their work. This is substantiated by Schaufeli et al. (2006, p. 702), who noted that dedication refers to being “strongly involved in one’s work, experiencing a sense of significance, enthusiasm, inspiration, pride and challenge”. Absorption, on the other hand, refers to a state of an employee’s full engrossment, concentration, and immersion in his/her work (Schaufeli et al., 2006). Drawing on the JD-R, mentoring and coaching serve as job resources that trigger the motivational process, thereby enhancing an employee’s work engagement. Higher levels of work engagement are reflected in increased energy, resilience, persistence, dedication, and involvement, as well as a willingness to go the extra mile, and are therefore negatively associated with quiet quitting. Previous research has found that work engagement is negatively related to withdrawal behaviours (Garg et al., 2021; Schaufeli et al., 2009), including quiet quitting (Xu et al., 2026). Therefore, it is hypothesised that work engagement is negatively related to quiet quitting.

**H3.**  *Work engagement is negatively related to the quiet quitting behaviours of graduate interns.*

The JD-R model provides a plausible mechanism that elucidates how work engagement mediates the relationship between mentoring and coaching and quiet quitting behaviours. Within the JD-R, work engagement serves as a motivational pathway that mediates the relationship between job resources and work outcomes (Bakker & Demerouti, 2017). Mentoring and coaching are job resources that activate the motivational process, resulting in higher levels of work engagement, which in turn reduces withdrawal behaviour such as quiet quitting. Thus, work engagement serves as the psychological mechanism through which mentoring and coaching is negatively associated with quiet quitting behaviours among graduate interns. Several studies have empirically supported the role of work engagement as a mediating mechanism between predictors and outcomes (Garg et al., 2021; Liu et al., 2024; Salanova & Schaufeli, 2008). In particular, a study by Garg et al. (2021) found that work engagement mediated the relationship between reverse mentoring and withdrawal behaviours. Therefore, based on the above discussion, we hypothesise the following:

**H4.**  *Work engagement mediates the negative relationship between perceived mentoring and coaching and quiet quitting behaviours of graduate interns.*

### 2.5. Job Insecurity

Job insecurity refers to ‘a perceived threat to the continuity and stability of employment as it is currently experienced’ (Shoss, 2017, p. 1914). Job insecurity has been categorised into qualitative and quantitative forms. Qualitative job insecurity refers to “the anticipation of losing valued job features” (Greenhalgh & Rosenblatt, 1984, p. 438) such as career opportunities, specific work tasks, and remuneration (De Witte et al., 2010; Vander Elst et al., 2014). On the other hand, quantitative job insecurity refers to “the perceived powerlessness to maintain desired continuity in a threatened job situation” (Greenhalgh & Rosenblatt, 1984, p. 441). In this study, the quantitative definition of job insecurity by Greenhalgh and Rosenblatt (1984) was adopted. Job insecurity was considered a moderator of the mediated relationship between work engagement and quiet quitting behaviours among graduate interns. Graduate intern employment is precarious and poses significant job stressors. In the JD-R model, job insecurity is conceptualised as a job demand and a job stressor (Bakker & Demerouti, 2007). Graduate interns face uncertainty about the continuity of their employment after the internship. Mseleku (2022) notes that the lack of employment in the South African labour market has precipitated post-internship graduate unemployment. Therefore, perceived job insecurity is a significant work stressor for graduate interns.

The JD-R boosting hypothesis proposes that excessive job demands heighten the positive impact of job resources on work engagement (Demerouti, 2025). As noted by Xanthopoulou et al. (2013, p. 76), “high job demands act as challenges, rendering the positive effect of resources on engagement more salient”. On the other hand, conservation of resources (COR) theory postulates that individuals have a strong inclination to protect and preserve their resources (conservation) and to avoid depletion, while simultaneously having a high propensity to acquire new resources (acquisition) (Hobfoll, 1989, 2011). The COR theory proposes that employees who face job insecurity, a job stressor for graduate interns in this study, are likely to engage in quiet quitting behaviours as a coping strategy to protect against further resource depletion. Therefore, the COR theory presents a competing view, proposing that high job insecurity may lead to resource depletion, thereby weakening rather than strengthening the positive pathway between mentoring and coaching and quiet quitting via work engagement. However, this study used the JD-R model and the boosting hypothesis to propose that job insecurity strengthens the relationship between mentoring and coaching via work engagement. This proposition is corroborated by Xanthopoulou et al. (2013), who found that self-efficacy (a personal resource) related positively to work engagement under conditions of high emotional and dissonance demands. Similarly, Dicke et al. (2018) found that self-efficacy, a personal resource, predicted work engagement under conditions of high job demands. Using the boosting hypothesis, we proposed that high job insecurity served as a significant job demand and stressor, strengthening the positive impact of mentoring and coaching on work engagement, which, in turn, is negatively associated with quiet quitting. Therefore, the following hypothesis is formulated:

**H5.**  *Perceived job insecurity moderates the indirect effect of mentoring and coaching on quiet quitting behaviour via work engagement, such that the negative indirect effect is stronger when job insecurity is high and weaker when job insecurity is low.*

**Figure 1:**
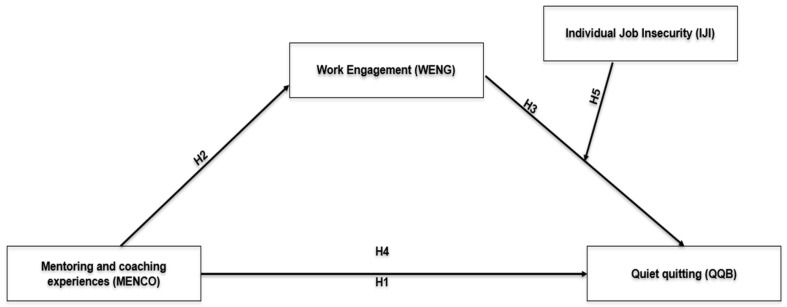
Theoretical model.

Based on the discussion above, the moderated mediation theoretical model of this study is depicted in Figure 1 below.

## 3. Methods and Procedure

This study is a quantitative study anchored in the positivist paradigm. A cross-sectional survey design was used; data collection for the independent, dependent, moderator, and mediator variables was conducted during the same time period (Hunziker & Blankenagel, 2024). Cross-sectional designs are susceptible to common method bias (Podsakoff & Todor, 1985) yet remain popular in management and organisational research (Spector, 2019).

The population of graduate interns targeted in this study predominantly comprised Generation Z employees entering the workforce in large numbers (Xueyun et al., 2023). Data were collected from 264 early-career Gen Z employees working in South African organisations as graduate interns in a fixed-term internship programme lasting 12 or 24 months. The age standard for this generation (1995–2010) indicates that the participants were 30 years old or younger. The graduate interns were recruited via LinkedIn, enabling the researchers to access a broader sampling pool in a cost-effective way. LinkedIn advertisements soliciting interns were posted, and invitations were sent to them. Thus, non-probability sampling methods, namely purposive and snowball sampling, were used to recruit graduate interns. Purposive sampling was used because the researchers targeted graduate interns currently employed in a fixed-term internship position. Graduate interns who participated in the study were encouraged to forward the invitation to other interns in their organisation or networks and were encouraged to like and repost the invitation so that it could reach more potential participants. Also, the researchers provided the study advertisement and survey link to their LinkedIn contacts and encouraged them to forward it to interns in their respective organisations and networks. In this way, snowball sampling helped to increase the sample of graduate interns who participated in the study.

A total of 271 graduate interns participated in the study, of whom 7 (2.58%) had missing values in their responses. The low level of missing data can be attributed to the survey design, which prompted participants to complete any unanswered items before submission, although incomplete responses were permitted. Given the small number of responses with missing data and the lack of a recognisable systematic pattern of missingness, listwise deletion was applied, resulting in a final sample of 264 for analysis. The majority of the 264 participants were females (61%); males comprised 38.3%. In total, 54.9% of the graduate interns held an honours degree, 34.8% held a bachelor’s degree, 3.4% held a master’s degree, and 6.8% held other qualifications. By racial group, 70.5% of the participants were black African, 10.6% were coloured, 10.2% were white, and 8.7% were Indian. The graduates were drawn from diverse organisational sectors, including financial services (20.8%), public service or government (11%), information technology and telecommunications (11.4%), manufacturing and engineering (12.1%), and retail and wholesale (8.7%). About 20% of the interns indicated the other organisational sector, while agriculture/forestry and fishing, chemical and petroleum, construction, mining, and transport had less than 5% representation. Most of the interns were employed in a 12-month fixed-term internship programme (71.2%), followed by a 24-month fixed-term internship programme (22.3%).

### Measures

The quiet quitting scale (Anand et al., 2024) was used to measure graduate interns’ quiet quitting behaviours. The quiet quitting scale measured the graduate intern’s level of disengagement, commitment, and desire to go above and beyond the minimum required deliverables. The scale consists of 8 items (e.g., I am doing the bare minimum work to avoid getting fired) and uses a 5-point Likert scale (1 = strongly disagree, 5 = strongly agree). In the current study, the scale demonstrated adequate psychometric properties, with a Cronbach’s alpha of 0.846. However, inspection of the ‘Item-Total Statistics’, specifically the ‘Corrected Item-Total Correlation’ statistics, indicated that item QQB1 was the only item with a value less than the recommended cut-off of 0.30 (Boateng et al., 2018), while others had values above 0.40. This indicated that the QQB1 score was not adequately related to the scores of the other quiet quitting scale items. Furthermore, ‘Cronbach’s alpha if Item Deleted’ statistics indicated that deleting item QQB1 (I often arrive late and leave early from work) would result in a modest increase in Cronbach’s alpha from 0.849 to 0.861. This item was flagged as potentially problematic but was retained pending further investigation. (In the CFA results, this item yielded a relatively low loading (0.313) and was removed. The re-run reliability analysis yielded a Cronbach’s alpha of 0.861).

The mentoring and coaching experiences of graduate interns were measured using the mentoring and coaching scale developed by Helber (2015). For this study, the graduate intern’s line manager was considered the primary mentor, and the questions were modified to reflect this and align with the target population of graduate interns. The scale consisted of 12 items measuring a unidimensional construct, with four facets: the career facet (e.g., my line manager gives me challenging assignments to help me develop my skills as a graduate intern), the psychosocial facet (e.g., my line manager is someone I can trust for advice and support), the vicarious facet (e.g., I work alongside my line manager when learning important tasks of the job), and the verbal persuasion facet (e.g., my line manager gives me verbal encouragement and feedback) (Helber, 2015). The mentoring and coaching scale yielded a Cronbach’s alpha of 0.956, which was acceptable and demonstrated reliability.

The Utrecht Work Engagement Scale (UWES-9) (Schaufeli et al., 2006) was used to measure work engagement in this study. Using nine items, this shortened scale measured the three dimensions of work engagement (vigour, dedication, and absorption): vigour (e.g., I am bursting with energy), dedication (e.g., I feel happy when I am working intensely), and absorption (e.g., I get carried away when I am working). All the items were rated using a 7-point Likert scale ranging from ‘never’ (0) to ‘always’ (6), with higher scores indicating higher levels of graduate intern engagement and lower scores indicating lower levels of graduate intern engagement. Although the Utrecht Work Engagement Scale (UWES-9) is considered a multidimensional scale, its three dimensions can be collapsed into a unidimensional measure (Schaufeli & Bakker, 2004). Several studies have supported the unidimensionality of the UWES-9 (Fong & Ho, 2015; Lindberg et al., 2025). The unidimensional UWES-9 in the current study demonstrated acceptable reliability (Cronbach’s alpha = 0.919).

Job insecurity was measured using the individual job insecurity scale developed by Låstad et al. (2015). The individual job insecurity scale measured the perceived graduate intern’s ability to maintain desired employment continuity after the internship ended. The scale consisted of three items that were modified to focus on graduate interns (e.g., “As a graduate intern, I feel insecure about the future of my job”). The job insecurity scale yielded a Cronbach’s alpha of 0.698, which marginally missed the recommended 0.70 cut-off (Nunnally & Bernstein, 1994). Item-total statistics were inspected to identify potentially problematic items. Inspection of the Corrected Item–Total Correlation statistics for the three items ranged from 0.462 to 0.565, which exceeded the recommended cut-off of 0.30 (Boateng et al., 2018) and indicated that the score of each item was related to the scores of other job insecurity items. The “Cronbach’s alpha if Item Deleted” statistics (IJI = 0.684; IJI2 = 0.596; IJ3 = 0.542) did not indicate that deletion of any of the scale items would result in an increase in the Cronbach’s alpha obtained. This indicated that all three adapted items contributed positively to the scale’s internal consistency; hence, they were retained. Thus, the adapted job insecurity scale appeared to demonstrate acceptable reliability, given that short-scale items tend to yield lower Cronbach’s alpha values (Pallant, 2016).

## 4. Results

The objective of this study was to evaluate the impact of line manager mentoring and coaching on quiet quitting behaviours among graduate interns. This study proposed that work engagement mediated this impact and that job insecurity moderated the mediated relationship, such that higher levels of job insecurity strengthened the indirect effects of mentoring and coaching on quiet quitting via work engagement. Common-method bias and confirmatory factor analysis were conducted as the initial data analysis, using SPSS 30 and AMOS graphics 30. Common method bias was assessed using Harman’s single-factor test, while all measures utilised in this study were subjected to confirmatory analysis. Confirmatory factor analysis was used to evaluate whether the items measured the indicators they were designed to measure and to assess the distinctiveness of the measures. Descriptive statistics, reliability analysis, and correlational analysis were conducted. Lastly, the SPSS PROCESS macro (Hayes & Little, 2022) was used to test the mediation and moderated mediation hypotheses proposed in this study.

Prior to the analysis, regression assumptions were assessed, and the results are reported in Table 1 below.

Table 1 indicates that most studentized residuals fell within the acceptable range of ±3; however, one observation (Case 54) exhibited a value of 4.15 and was flagged as a potential outlier. The Bonferroni corrected test indicated that this outlier was statistically significant (*p* < 0.05). This was concerning but did not imply that the model was invalid. The assessment of influential observations indicated that the DFBETA ranged from −0.15 to 0.04, which was below the recommended cut-off range (>2/√n or >1), indicating that despite a significant detectable outlier, there was no observation that had a “demonstrably larger impact on the calculated values of various estimates (coefficients, standard errors, t-values, etc.) than is the case for most of the other observations” (Belsley et al., 2005, p. 11). Thus, the outlier did not materially affect the regression estimates. Normality was assessed using skewness and kurtosis; skewness values ± 2 and kurtosis values ± 7 were considered acceptable (H.-Y. Kim, 2013; Kline, 2015). The skewness value of 0.52 and the kurtosis value of 1.05 indicate mild positive skewness and mild kurtosis, respectively, and both fell within commonly acceptable ranges, suggesting that the residuals approximate normality. Multicollinearity was assessed using the variance inflation factor (VIF), with values below 5 considered acceptable and tolerance with values greater than 0.2 also considered acceptable (Hair et al., 2021). The VIF values ranged from 1.07 to 2.06, while the tolerance values ranged from 0.49 to 0.94, demonstrating that multicollinearity was not an issue and did not bias the estimate of the interaction term. Lastly, the Breusch–Pagan test (both Normal and Robust) was not significant, indicating that homoscedasticity was supported. Taken together, the presence of an outlier was concerning, but because it was not influential, it did not bias the estimates and seemed theoretically substantive within the JD-R model; thus, the outlier was retained.

The other initial analysis involved assessing the risk of common method bias using Harman’s single-factor test, computed via exploratory factor analysis (EFA). The results of Harman’s single-factor test indicated that common method variance did not pose a serious threat in this study, as the total variance explained by the single factor fell below the recommended cut-off of 50%. Descriptive statistics, reliability, and correlation analysis were conducted, and the results are discussed below. Next, the mentoring and coaching scale, work engagement scale, quiet quitting scale, and job insecurity scale were subjected to confirmatory factor analysis using Amos to assess whether the scale items measured the constructs they were intended to reflect and to ascertain the distinctiveness of the measures.

### 4.1. Descriptive Statistics, Reliability Analysis, and Correlational Analysis

Descriptive statistics, reliability analysis, and correlational analysis were conducted, and the results are depicted in Table 2. Mentoring and coaching yielded a moderate to high mean (M = 3.731, SD = 0.930), followed by work engagement (M = 4.183, SD = 1.006), and job insecurity (M = 3.848, SD = 0.855), while quiet quitting had the lowest mean (M = 2.183, SD = 0.774), Table 1 also shows that mentoring and coaching was positively related to work engagement (r = 0.695, *p* < 0.01) and negatively related to quiet quitting (r = −0.661, *p* < 0.01) and job insecurity (r = −0.231, *p* < 0.01). Work engagement was negatively related to quiet quitting (r = −0.702, *p* < 0.01) and job insecurity (r = −0.228, *p* < 0.01), while job insecurity was positively related to quiet quitting (r = 0.302, *p* < 0.01). In terms of reliability analysis, the mentoring and coaching scale (0.956), work engagement scale (0.919), and quiet quitting scale (0.849) demonstrated adequate psychometric properties and yielded Cronbach’s alpha values that met the recommended cut-off of 0.70 (Nunnally, 1978). The job insecurity scale yielded a Cronbach’s alpha of 0.698, which is acceptable given its three-item structure (Pallant, 2016).

### 4.2. Confirmatory Analysis

Confirmatory factor analysis was conducted using IBM SPSS Amos 30 Graphics. Item QQB1 (I often arrive late and leave early from work) was excluded because it yielded a borderline factor loading of 0.313. During item analysis, item QQB1 was flagged as potentially problematic because it exhibited a Corrected Item–Total Correlation less than the recommended cut-off value of 0.30, which indicated that it made a limited contribution to the reliability of the quiet quitting scale (Boateng et al., 2018) and resulted in a modest increase in Cronbach’s alpha if the item were deleted. In the confirmatory factor analysis, the item yielded a relatively low factor loading (0.313), below the preferred 0.05 for practical significance (Hair et al., 2021), indicating a weak association with the quiet quitting construct. It was decided to exclude item QQB 1 prior to re-estimating the measurement model. Table 3 shows that the four-factor model demonstrated a better fit, with X^2^/df < 3 and the Comparative Fit Index (CFI) > 0.90, Tucker–Lewis Index (TLI) > 0.90, and Incremental Fit Index (IFI) > 0.90, all of which exceeded the recommended cut-off of 0.90 (Bentler & Bonett, 1980) but did not meet the stringent cut-off of 0.95 (Hu & Bentler, 1999). Furthermore, the Standardised Root Mean Squared Residual (SRMR) was 0.053, which demonstrated a good model fit, and the Root Mean Square Error of Approximation (RMSEA) had a value of 0.066, which demonstrated a good-to-reasonable fitting model (Browne & Cudeck, 1993; Jöreskog & Sörbom, 1993).

### 4.3. Convergent and Discriminant Validity

The standardised factor loadings, average variance extracted (AVE), and composite reliability (CR) are shown in Table 4. Table 4 shows that all factor loadings were greater than the recommended 0.5 (Hair et al., 2021), and the composite reliability values for the constructs in this study were greater than the recommended value of 0.70 (Fornell & Larcker, 1981). The AVE values for mentoring and coaching (0.654), work engagement (0.572), and quiet quitting (0.508) exceeded the recommended value of 0.5 (Fornell & Larcker, 1981), indicating adequate convergent validity. The AVE value for job insecurity (0.448) was slightly below the recommended cut-off, suggesting marginal convergent validity, even though the CR value was above 0.7 and factor loadings were above 0.50. Researchers have indicated that AVE values slightly below the 0.50 cut-off, accompanied by composite values greater than 0.7, may still be considered acceptable indicators of convergent validity (Fornell & Larcker, 1981; Gallardo-Vázquez et al., 2020; C.-C. Huang et al., 2013).

Furthermore, discriminant validity was assessed using the Fornell and Larcker (1981) criterion, which stipulates that discriminant validity is supported when the square root of each construct’s AVE is greater than its correlations with other constructs. Table 2 shows that discriminant validity was supported, as the correlations among variables were lower than the square roots of each variable’s AVE.

### 4.4. Direct Effects and Mediation Effects

A simple mediation analysis was conducted using the SPSS PROCESS macro model 4 to test whether work engagement mediated the relationships between mentoring and coaching and quiet quitting behaviours among graduate interns. Table 5 shows that mentoring and coaching had a significant, direct negative effect on graduate interns’ quiet quitting behaviour (β = −0.336, *p* < 0.001), thereby supporting hypothesis 1. Table 5 also shows that mentoring and coaching had a significant and positive direct effect on work engagement (β = 0.695, *p* < 0.001), thereby supporting hypothesis 2. Work engagement had a significant, negative direct effect on quiet quitting behaviours among graduate interns (β = −0.468, *p* < 0.001), thereby confirming hypothesis 3.

The mediation hypothesis results are also shown in Table 5. Table 5 shows that mentoring and coaching had a significant negative total effect in the absence of work engagement (β = −0.551, *p* < 0.001) and a direct effect on quiet quitting (β = −0.280, *p* < 0.001), and the indirect effects of mentoring and coaching on quiet quitting via work engagement was significant (β = −0.271, SE = 0.044, 95% CI: −0.347 to −0.170), thereby supporting hypothesis 4. The indirect effect was significant because the 95% confidence interval did not include zero. Thus, work engagement plays an important role in partially mediating the relationship between line manager mentoring and coaching and graduate interns’ quiet quitting behaviours.

### 4.5. Moderation Analysis

Table 6 shows that the interaction between work engagement and job insecurity was significant (β = −0.098, *p* < 0.01), indicating that job insecurity moderated the relationship between work engagement and quiet quitting among graduate interns. The last step involved testing the moderated mediation hypothesis using the SPSS PROCESS macro (model 14). Hypothesis 6 proposed that job insecurity moderates the indirect effect of mentoring and coaching on quiet quitting behaviour via work engagement, such that the negative indirect effect is stronger when job insecurity is high and weaker when job insecurity is low.

### 4.6. Moderated Mediation

Table 7 below shows the bootstrapping results for the conditional indirect effects of mentoring and coaching on quiet quitting via work engagement at different levels of job insecurity.

**Figure 2:**
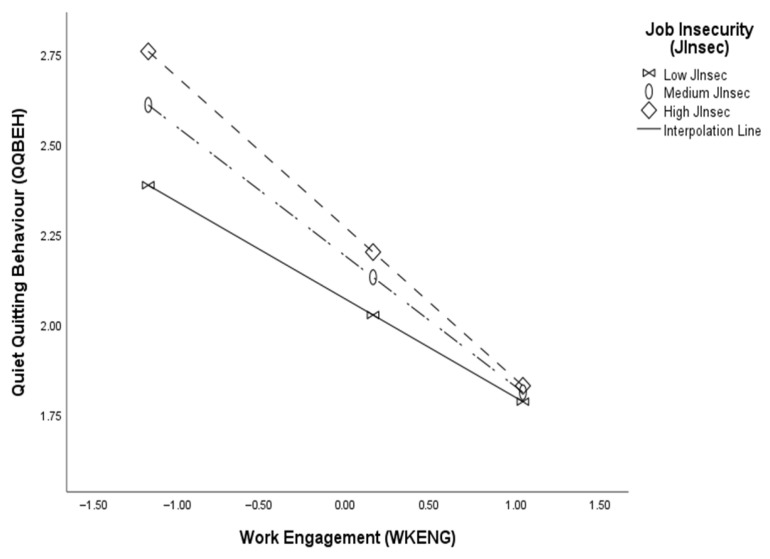
Moderating effect of job insecurity on the indirect effect of mentoring and coaching on quiet quitting through work engagement.

Table 7 shows that the conditional indirect effect of coaching and mentoring on quiet quitting through work engagement was significantly negative and stronger under high job insecurity (effect = −0.315, bootSE = −0.048, 95% bootstrap CI: [−0.399, −0.210]), significantly negative and moderate under moderate job insecurity (effect = −0.271, bootSE = −0.044, 95% bootstrap CI: [−0.343, −0.172]), and significantly negative and lower under conditions of low job insecurity (effect = −0.204, bootSE = −0.049, 95% bootstrap CI: [−0.288, −0.095]). Figure 2 below shows that a steeper negative slope at high levels of job insecurity indicates that the negative effect of work engagement on quiet quitting is stronger under those conditions. In addition, the index of moderated mediation, which indicates that “any two conditional indirect effects estimated at different values of the moderator are significantly different from one another” (Hayes, 2015, p. 2), was significant (Index = 0.067, bootSE = −0.024, 95% bootstrap CI: [−0.116, −0.019]), thus supporting hypothesis 5. Thus, the results confirm hypothesis 5 and demonstrate that high job insecurity strengthens the indirect negative relationship between mentoring and coaching and quiet quitting via work engagement, and lower levels of job insecurity weaken the relationship. This study supported the boosting hypothesis by showing that high job insecurity strengthened the negative relationship between work engagement and quiet quitting, which was associated with a stronger indirect effect of mentoring and coaching on withdrawal behaviour through work engagement.

## 5. Conclusions

### 5.1. Discussion

This study investigated the impact of mentoring and coaching on graduate interns’ quiet quitting behaviours. Furthermore, work engagement was a key mediator in this relationship, and job insecurity moderated this mediated relationship. The study showed that mentoring and coaching have a positive impact on graduate interns’ work engagement and a negative impact on quiet quitting behaviours. These results dovetail with the social exchange theory (SET) (Cropanzano & Mitchell, 2005) and the Job Demands–Resources model (Bakker & Demerouti, 2007). Using social exchange theory, graduate interns who receive high-quality mentoring and coaching are likely to feel obligated and indebted to reciprocate by demonstrating higher levels of engagement and commitment, which are negatively associated with quiet quitting behaviours. Previous research has shown that mentoring and coaching increase employees’ work engagement (Ladyshewsky & Taplin, 2018; F. Yang et al., 2022; Z. Wang et al., 2018), which in turn reduces quiet quitting behaviours. Also, drawing from the Job Demands–Resources model (Demerouti et al., 2001), mentoring and coaching are key job resources that trigger the motivational process, resulting in increased work engagement and reduced quiet quitting behaviours among graduate interns. Job resources such as social support, supervisor support, and organisational support were inversely related to quiet quitting (Anand et al., 2024; Gün et al., 2025). In line with previous research, this study found that line manager mentoring and coaching are inversely related to quiet quitting behaviours among graduate interns.

Furthermore, this study found that higher levels of perceived job insecurity were positively associated with quiet quitting, and job insecurity moderated the mediating role of work engagement in the relationship between mentoring and coaching and quiet quitting. In line with social exchange theory, higher levels of job insecurity may trigger graduate interns’ negative reciprocation, which in turn results in quiet quitting behaviours. This study found a positive relationship between job insecurity and quiet quitting. Previous research has shown that job insecurity is positively associated with quiet quitting behaviour (Azizah et al., 2025; Lestari et al., 2024). More interestingly, this study supported the JD-R’s boosting hypothesis, which posits that higher levels of perceived job insecurity strengthen the impact of job resources on work engagement (Bakker & Demerouti, 2007). Higher levels of perceived job insecurity among graduate interns strengthened the positive impact of mentoring and coaching on work engagement, which in turn was negatively associated with quiet quitting. This corroborates the boosting hypothesis of the JR-D model, which has been supported in previous research as well (Dicke et al., 2018; Xanthopoulou et al., 2013).

This study contributes to the literature on quiet quitting among early-career Gen Z employees entering the world of work, characterised by turbulence, uncertainty, novelty, and ambiguity (TUNA). The world of work is still adapting to the drastic changes ushered in by the COVID-19 pandemic. There is a paucity of studies that have explored factors that can minimise quiet quitting (Burch et al., 2025; Karrani et al., 2025), and this study positions mentoring and coaching as a plausible intervention to minimise quiet quitting among early-career Gen Z employees. The results of this study confirm that mentoring and coaching drive work engagement and motivation and are negatively related to quiet quitting behaviours among graduate interns. Manager behaviours such as negative supervisor gossiping (Šimáček et al., 2026), bullying (Galanis et al., 2024), and abusive supervision (Bhawna et al., 2026; Gong et al., 2026) increased the quiet quitting behaviours of subordinates. This emphasises the role of managers in mitigating or increasing quiet quitting behaviours among their subordinates.

Given the increase in quiet quitting behaviours among Gen Z employees (Oktavia et al., 2025; Srivastava & Saxena, 2025), mentoring and coaching can serve as developmental tools that drive the engagement and commitment of graduate interns. Organisations can mitigate the challenge of quiet quitting among early-career Gen Z employees by providing adequate mentoring and coaching. Mentoring and coaching are key drivers of work engagement (Ladyshewsky & Taplin, 2018; F. Yang et al., 2022), self-esteem, self-efficacy (Ghosh et al., 2012; Moon et al., 2025; Sloan et al., 2025), employee retention (Kissoonduth & Webb, 2021), and career growth (Gong et al., 2022). Furthermore, organisations that equip managers to mentor and coach early-career Gen Z employees are likely to enhance managers’ job satisfaction, work engagement, organisational commitment, and job performance (Ghosh & Reio, 2013). Thus, mentoring and coaching mutually benefit both the mentee/coachee and the mentor/coach.

This study makes a theoretical contribution by combining social exchange theory (SET) (Cropanzano & Mitchell, 2005) and the Job Demands–Resources (JD-R) model (Bakker & Demerouti, 2007) as theoretical frameworks that explain how manager mentoring and coaching is positively associated with work engagement and negatively related to quiet quitting among graduate interns who face high levels of job insecurity. By integrating social exchange theory and the Job Demands–Resources model, this study provided a plausible psychological mechanism to explain why mentoring and coaching negatively predict quiet quitting and why work engagement plays a key mediating role, while job insecurity moderates the mediated relationship. Hence, this study contributes by identifying job insecurity among graduate interns as a contextual moderator. The indirect effects of coaching and mentoring on quiet quitting via work engagement depend on the contextual moderator of job insecurity. Extending this contribution, this study showed that higher levels of job insecurity typical of temporary employment, such as internships, increased the salience of mentoring and coaching for graduates’ work engagement. Higher levels of job insecurity strengthened the indirect impact of mentoring and coaching on graduate quiet quitting via work engagement, thereby supporting the JD-R’s boosting hypothesis (Bakker & Demerouti, 2007).

Furthermore, this study makes a theoretical contribution by expanding the nomological network of mentoring and coaching, linking them as developmental tools that can mitigate quiet quitting among early-career Gen Z employees. Quiet quitting is on the rise in contemporary organisations and particularly among Gen Z employees (Oktavia et al., 2025; Srivastava & Saxena, 2025). Although previous research has explored the outcomes of mentoring and coaching, such as work engagement (Tanskanen et al., 2019; L.-y. Wang et al., 2024), organisational commitment (S. Kim, 2014), in-role and extra-role behaviours (J. T. Huang & Hsieh, 2015; S. Kim & Kuo, 2015), job satisfaction, performance (S. Kim, 2014; Pousa et al., 2017), and employee well-being (Zhao & Liu, 2020), there is a paucity of studies that have linked mentoring and coaching to quiet quitting. Hence, this study contributes to the mentoring and coaching literature by linking quiet behaviour as an outcome of mentoring and coaching.

### 5.2. Limitations and Future Directions of the Study

This research proffers a plausible theoretical framework to explain why line manager mentoring and coaching negatively predicted the quiet quitting of graduate interns by integrating the social exchange theory (SET) and Job Demands–Resources model (JD-R). Furthermore, the social exchange theory and the Job Demands–Resources model explained the psychological mechanisms through which work engagement mediated the relationship between mentoring and coaching and quiet quitting, and why job insecurity moderated the mediated relationship. Despite this study’s useful insights, the following limitations are discussed.

This study used a cross-sectional design, which has limitations because it precludes the inferring of causal relationships. This limitation creates an opportunity to use longitudinal research to examine how mentoring and coaching can have long-term effects on quiet quitting and on increasing graduate interns’ work engagement throughout the internship. Longitudinal designs are superior compared to cross-sectional designs because they are better at ‘suggesting’ causality and are less susceptible to common method bias compared to cross-sectional research designs (Maxwell & Cole, 2007). Furthermore, this study used self-report measures, which are susceptible to social desirability effects and common method bias (Podsakoff et al., 2003). Even though we conducted a Harman single-factor test to assess the risk of common method variance, the use of self-report measures presents a limitation. A mixed-methods research design is recommended for future research because it employs quantitative and qualitative methods and yields multiple data sources that can be used with self-report measures, thereby enhancing triangulation.

Using the LinkedIn platform enabled researchers to reach participants and their networks by encouraging participants to share the survey with their networks or to recommend other participants who met the criteria (Chenane & Hammond, 2022). However, this can pose a limitation as participants could share the invitation and survey link with other potential participants in their networks, potentially leading to the over-representation of networks that did not reflect the broader graduate intern population (Kukafka et al., 2022). Furthermore, participants could share the invitation and survey link with potential participants who shared their characteristics, thereby reducing heterogeneity and variability in the sample.

Furthermore, in the South African context, graduate interns come from diverse socio-economic backgrounds, and graduates from privileged backgrounds are more likely to be active LinkedIn members than graduate interns from lower socio-economic backgrounds. Graduates from less privileged backgrounds are likely to lag behind in Internet skills and experience, which might limit their active participation on the LinkedIn platform (Hargittai, 2020). Therefore, the limitation of using LinkedIn as a platform to recruit graduate interns for this study is the oversampling of those with privileged socioeconomic backgrounds and undersampling of those with underprivileged backgrounds. This may have biased the sample and limited the generalisability of the results.

Another limitation of this study lies in the use of non-probability sampling methods, namely purposive and snowball sampling. Non-probability sampling methods do not give everyone in the research population an equal chance or probability of being selected into the sample, and only available and willing participants are likely to be included (Saunders et al., 2023). In contrast, probability sampling methods give everyone in the research population an equal chance of being selected into the sample; hence, they yield a representative sample of the population (Gravetter & Forzano, 2012). The non-probability sampling methods, including purposive and snowball sampling, used in this study yielded unrepresentative samples that do not fully reflect the characteristics of the research population, thereby limiting the generalisability of the results. Furthermore, the sample consisted of a subgroup of Gen Z employees in precarious positions and, therefore, the study is limited in its generalisability to the broader Gen Z cohort.

Lastly, the AVE of the job insecurity scale (0.448) fell slightly below the recommended 0.50 (Fornell & Larcker, 1981), which is a limitation of this study. The job insecurity scale was a three-item scale, which limited the amount of shared variance captured by the construct. The job insecurity scale yielded moderate factor loadings (0.584, 0.667, and 0.748), which exceeded the minimum acceptable cut-off of 0.50 (Hair et al., 2021), suggesting that the items contributed to explaining job insecurity. The scale yielded a composite reliability above 0.70, indicating acceptable internal consistency (Fornell & Larcker, 1981). Also, researchers have indicated that an AVE slightly below 0.50 may be acceptable when combined with a composite reliability above 0.70 (Fornell & Larcker, 1981; Gallardo-Vázquez et al., 2020; C.-C. Huang et al., 2013; Lam, 2012). Additionally, discriminant validity was established, as the square roots of the AVEs exceeded the inter-construct correlations. Thus, items were retained to preserve the construct’s content validity. This limitation presents an opportunity for future researchers to refine the scale, particularly in contexts involving early-career employees in precarious employment settings, such as graduate interns.

A fruitful avenue of research is to explore how different leadership styles affect quiet quitting. Future studies can examine how leadership styles affect followers’ quiet quitting behaviours. For example, Burch et al. (2025) conceptually explored this avenue and found that authentic leadership fostered a supportive and engaging work environment and was negatively related to quiet quitting. Future studies should explore how follower-centred leadership styles (servant leadership, transformational leadership, humble leadership, participative leadership, empowering leadership) and destructive leadership (abusive supervision and toxic leadership) mitigate or heighten quiet quitting behaviours among employees, or how they moderate the relationship between mentoring and coaching and quiet quitting.

Future studies can also explore the black box of quiet quitting in research by exploring mediators and moderators. Research on quiet quitting has focused on literature reviews, antecedents, and outcomes of quiet quitting (Alhammadi et al., 2025; Gone et al., 2026; Nguyen & Vu, 2025; Srivastava et al., 2024; Taufik et al., 2024; Xueyun et al., 2023), and very few studies have explored the mediation and moderators. For example, a study by Karrani et al. (2025) included work alienation as a mediator and inclusive leadership as a moderator between relational job design and quiet quitting. Also, K. T. Kim and Sohn (2024) included job satisfaction and affective commitment as mediators of the relationship between quiet quitting and intention to quit, and psychological safety as a moderator of the mediation relationships. Future studies can explore possible mediators and moderators such as organisational culture, leader–member exchange, personality traits, and individual differences.

## Figures and Tables

**Table 1:** Regression diagnostics and assumption check.

Assumption	Diagnostic	Result	Criteria/Cut-Off	Assessment
Outliers	Standardised residuals	Min = −2.57; Max = 4.15	|t| > 3	One outlier (Case 54)
	Bonferroni-adjusted test	*p* = 0.012	*p* < 0.05	Significant
Normality	Skewness	0.52	|skew| ≤ 2	Acceptable
	Kurtosis	1.05	|kurtosis| ≤ 7	Acceptable
Influential Observation	Maximum DFBETA	≤0.05	>2/√n or >1	No influential observation
Multicollinearity	VIF range	1.07–2.06	VIF > 5–10	Low
	Tolerance range	0.49–0.94	<0.20	Low
Homoscedasticity	Breusch–Pagan test	χ^2^ (4) = 5.50, *p* = 0.24	*p* < 0.05	Supported
	Robust Breusch–Pagan	χ^2^ (4) = 3.67, *p* = 0.46	*p* < 0.05	Supported

**Table 2:** Descriptive statistics, reliability, and correlations analysis.

Scale	M	SD	α	1	2	3	4
Mentoring and Coaching	3.731	0.930	0.956	(0.809)			
Work Engagement	4.183	1.006	0.919	0.695 **	(0.756)		
Quiet Quitting	2.183	0.774	0.849	−0.661 **	−0.702 **	(0.713)	
Job Insecurity	3.848	0.855	0.698	−0.231 **	−0.228 **	0.302 **	(0.670)

N = 264: SD = standard deviation; α = Cronbach’s alpha; ** *p* < 0.01. The values on the diagonal represent the square root of AVE.

**Table 3:** Confirmatory factor analysis.

Measurement Model	*X* ^2^	*Df*	*X* ^2^ */df*	CFI	TLI	IFI	SRMR	RMSEA
Four-factor model	987.654	428	2.308	0.905	0.906	0.901	0.053	0.066
Three-factor model	1106.295	431	2.567	0.886	0.877	0.887	0.061	0.077
Two-factor model	1737.368	433	4.012	0.780	0.763	0.781	0.082	0.107
Single-factor model	1964.502	434	4.527	0.742	0.723	0.743	0.085	0.116

N = 264. Four-factor model: mentoring and coaching; work engagement; quiet quitting; job insecurity. Three-factor model: mentoring and coaching; work engagement; quiet quitting + job insecurity. Two-factor model: mentoring and coaching + work engagement; quiet quitting + job insecurity. Single factor: All constructs merged into a single factor.

**Table 4:** Standardised factor loadings, average variance extracted (AVE), and composite reliability (CR).

Construct	Item	Item Wording	Standardised Factor Loading	AVE	CR
Job Insecurity	IJI1	IJI1: As a graduate intern, I am worried that I will have to leave my job before I would like to.	0.584	0.448	0.707
	IJI2	IJI2: I worry about keeping my job after the internship is complete.	0.667		
	IJI3	IJI3: I am afraid I may lose my job in the near future	0.748		
Work Engagement	WENG1	WE1: At my work, I feel bursting with energy.	0.668	0.572	0.921
	WENG2	WE2: My job inspires me.	0.849		
	WENG3	WE3: I get carried away when I am working.	0.518		
	WENG4	WE4: I feel happy when I am working intensely.	0.617		
	WENG5	WE5: At my job, I feel strong and vigorous	0.749		
	WENG6	WE6: When I get up in the morning, I feel like going to work.	0.776		
	WENG7	WE7: I am proud of the work that I do.	0.840		
	WENG8	WE8: I am immersed in my job.	0.837		
	WENG9	WE9: I am enthusiastic about my job.	0.871		
Quiet Quitting	QQB2	QQB2: I often avoid working more hours if there is no additional pay	0.643	0.508	0.877
	QQB3	QQB3: I am doing the bare minimum work to avoid being fired.	0.611		
	QQB4	QQB4: I feel there is a lack of opportunities to learn and grow in my organisation.	0.751		
	QQB5	QQB5: I feel there is a lack of meaningfulness at work.	0.845		
	QQB6	QQB6: I feel I have a lack of interest in attending meetings.	0.698		
	QQB7	QQB7: I feel there is a lack of passion and enthusiasm in me to work above and beyond.	0.718		
	QQB8	QQB8: I feel there is a lack of feeling regarding my employer’s caring for me.	0.698		
Mentoring and Coaching	MENCO1	MENCO1: My line manager coaches and guides me in understanding my role as a graduate intern.	0.889	0.654	0.958
	MENCO2	MENCO2: My line manager helps to sponsor the development and advancement in my career	0.834		
	MENCO3	MENCO3: My line manager is someone I could trust for advice and support.	0.871		
	MENCO4	MENCO4: I would consider my line manager to be a trusted friend	0.706		
	MENCO5	MENCO5: My line manager provides counselling advice to me	0.799		
	MENCO6	MENCO6: My line manager demonstrated the appropriate behaviours and skills that I use in my job	0.855		
	MENCO7	MENCO7: I worked alongside my line manager while learning the tasks of the job.	0.737		
	MENCO8	MENCO8: My working style is very similar to my line manager	0.659		
	MENCO9	MENCO9: My line manager gives me verbal encouragement and feedback.	0.853		
	MENCO10	MENCO10: My line manager has the knowledge and skills to help guide my development and growth	0.826		
	MENCO11	MENCO11: I consider my line manager as a skilled mentor and coach in his/her position.	0.888		
	MENCO12	MENCO12: My line manager gives me challenging assignments to help develop my skills	0.752		

**Table 5:** Direct effects and mediation effects.

PATH IV → DV	β	SE	t	*p*	LL95%CI	UL95%CI
H1: MENCOA → QQB	−0.336	0.48	−5.813	<0.001	−0.374	−0.1850
H2: MENCOA → WENG	0.695	0.48	15.661	<0.001	0.658	0.847
H3: WENG → QQB	−0.468	0.44	−8.106	<0.001	−0.448	−0.273
**Total Direct and Indirect Effects of MENCOA on QQB**						
	**Effect**	**SE**	**t**	***p***	**LL95%CI**	**UL95%CI**
Total effects	−0.551	0.39	−14.267	<0.001	−0.627	−0.475
Direct effects	−0.280	0.48	−5.813	<0.001	−0.374	−0.185
Indirect effects	−0.271	0.44	-	-	−0.347	−0.170

N = 264: Notes: MENCOA = mentoring and coaching; QQB = quiet quitting behaviour; WENG = work engagement; β = standardised coefficient; SE = standard error; LL95%CI = lower level of 95% confidence interval; UL95%CI = upper level of 95% confidence interval.

**Table 6:** Moderation effects.

Moderation Effects: DV → QQB					
Variables	B	SE	t	LL95%CI	UL95%CI
Constant	3.061	0.186	16.500	2.695	3.426
MANCOA	−0.240 *** (−0.288)	0.049	−4.943	−0.335	−0.144
WENG	−0.346 *** (−0.450)	0.044	−7.931	−0.432	−0.260
JInsec	0.118 ** (0.131)	0.038	3.116	0.044	0.193
WENG xJInsec	−0.089 * (−0.098)	0.039	−2.298	−0.164	−0.013

N = 264: Notes: MENCOA = mentoring and coaching; WENG = work engagement; B = unstandardised coefficient (standardised coefficient in brackets); SE = standard error; LL95%CI = lower level of 95% confidence interval; UL95%CI = upper level of 95% confidence interval; * *p* < 0.05, ** *p* < 0.01, *** *p* < 0.001 (two-tailed).

**Table 7:** Conditional indirect effects at levels of job insecurity.

JInsec		MENCOA → WENG → QQB		
	Effect	BootSE	BootLLCI	BootULCI
M − 1SD (−0.848)	−0.204	0.049	−0.288	−0.095
M (0.152)	−0.271	0.044	−0.343	−0.172
M + 1SD (0.818)	−0.315	0.048	−0.399	−0.210
Moderated mediated index	Index	BootSE	BootLLCI	BootULCI
	−0.067	0.024	−0.116	−0.019

N = 264: Notes: MENCOA = mentoring and coaching; QQB = quiet quitting behaviour; WENG = work engagement; JInsec = job insecurity; BootSE = standard error; BootLL95%CI = lower level of 95% confidence interval; BootUL95%CI = upper level of 95% confidence interval.

## Data Availability

The data that support the findings of this study are available from the corresponding author, Cebile Tebele, upon reasonable request.
